# Identification of potential drug targets for allergic diseases from a genetic perspective: A mendelian randomization study

**DOI:** 10.1002/clt2.12350

**Published:** 2024-04-04

**Authors:** Hui Wang, Jianyu Pang, Yuguan Zhou, Qi Qi, Yuheng Tang, Samina Gul, Miaomiao Sheng, Juhua Dan, Wenru Tang

**Affiliations:** ^1^ Laboratory of Molecular Genetics of Aging & Tumor Medicine School Kunming University of Science and Technology Kunming Yunnan China

**Keywords:** allergic diseases, mendelian randomization, protein, targeted therapy

## Abstract

**Background:**

Allergic diseases typically refer to a heterogeneous group of conditions primarily caused by the activation of mast cells or eosinophils, including atopic dermatitis (AD), allergic rhinitis (AR), and asthma. Asthma, AR, and AD collectively affect approximately one‐fifth of the global population, imposing a significant economic burden on society. Despite the availability of drugs to treat allergic diseases, they have been shown to be insufficient in controlling relapses and halting disease progression. Therefore, new drug targets are needed to prevent the onset of allergic diseases.

**Method:**

We employed a Mendelian randomization approach to identify potential drug targets for the treatment of allergic diseases. Leveraging 1798 genetic instruments for 1537 plasma proteins from the latest reported Genome‐Wide Association Studies (GWAS), we analyzed the GWAS summary statistics of Ferreira MA et al. (nCase = 180,129, nControl = 180,709) using the Mendelian randomization method. Furthermore, we validated our findings in the GWAS data from the FinnGen and UK Biobank cohorts. Subsequently, we conducted sensitivity tests through reverse causal analysis, Bayesian colocalization analysis, and phenotype scanning. Additionally, we performed protein‐protein interaction analysis to determine the interaction between causal proteins. Finally, based on the potential protein targets, we conducted molecular docking to identify potential drugs for the treatment of allergic diseases.

**Results:**

At Bonferroni significance (*p* < 3.25 × 10^−5^), the Mendelian randomization analysis revealed 11 significantly associated protein‐allergic disease pairs. Among these, the increased levels of TNFAIP3, ERBB3, TLR1, and IL1RL2 proteins were associated with a reduced risk of allergic diseases, with corresponding odds ratios of 0.82 (0.76–0.88), 0.74 (0.66–0.82), 0.49 (0.45–0.55), and 0.81 (0.75–0.87), respectively. Conversely, increased levels of IL6R, IL1R1, ITPKA, IL1RL1, KYNU, LAYN, and LRP11 proteins were linked to an elevated risk of allergic diseases, with corresponding odds ratios of 1.04 (1.03–1.05), 1.25 (1.18–1.34), 1.48 (1.25–1.75), 1.14 (1.11–1.18), 1.09 (1.05–1.12), 1.96 (1.56–2.47), and 1.05 (1.03–1.07), respectively. Bayesian colocalization analysis suggested that LAYN (coloc.abf‐PPH4 = 0.819) and TNFAIP3 (coloc.abf‐PPH4 = 0.930) share the same variant associated with allergic diseases.

**Conclusions:**

Our study demonstrates a causal association between the expression levels of TNFAIP3 and LAYN and the risk of allergic diseases, suggesting them as potential drug targets for these conditions, warranting further clinical investigation.

## 1 INTRODUCTION

Allergic diseases usually refer to a group of heterogeneous diseases mainly caused by mast cell or eosinophil activation, including atopic dermatitis (AD), allergic rhinitis (AR) and asthma.^1^ The pathogenesis of allergic diseases remains unclear, likely arising from the complex interplay of various factors such as genetics, environment, microbiota, and immune system function. However, these allergic diseases share common underlying mechanisms, partially mediated by immunoglobulin E.^1^ The incidence of asthma and other allergic diseases (such as AR and AD) has increased significantly in recent decades. These conditions affect approximately one‐fifth of the global population,^2^ posing a substantial burden worldwide. Asthma is a chronic inflammatory respiratory disease, which is characterized by increased sensitivity and respiratory obstruction. Its symptoms include dyspnea, coughing, chest tightness and wheezing. Severe asthma accounts for 5%–10% of the asthmatic population in the world. Unfortunately, about 3.6% of patients with asthma still experience poor asthma control even when receiving standard treatment.^3^ AD is a common inflammatory skin disease, which is characterized by recurrent eczema‐like lesions and strong itching. It affects individuals of all ages and ethnicities, causing significant psychosocial impacts on both patients and their relatives. Atopic dermatitis is the main cause of global burden of skin diseases.^4^ AR is a common chronic upper respiratory disease, which is characterized by nasal mucosal inflammation caused by allergens. It usually shows symptoms such as stuffy nose, runny nose, sneezing and itchy nose.^5^ These symptoms significantly impact the quality of life for affected individuals, imposing a substantial burden on both their families and society at large.^6^ Research suggests that allergic diseases (asthma, AR, and AD) coexist to a certain extent due to the presence of numerous shared genetic risk variants. These variants contribute to alterations in the expression of immune‐related genes.^7^ Although there are some drugs for allergic diseases, such as fluticasone,^8^ salmeterol,^9^ loratadine,^10^ dupilumab,^11^ and hydrocortisone.^12^ However, due to the high prevalence of allergic diseases and the drawbacks of existing treatment medications, such as significant side effects, incomplete symptom relief, and the need for long‐term maintenance therapy, research and development in the field of allergic disease medications remain urgent.

Human proteins play a crucial role in the onset and progression of diseases and represent the primary category of drug targets.^13^ Research indicates that proteins associated with genetic links to diseases have significantly increased chances of gaining market approval as drug targets.^14^ Mendelian randomlization (MR) is a genetic method that utilizes Single Nucleotide Polymorphisms (SNPs) from Genome‐Wide Association Studies (GWAS) as genetic instruments to estimate the causal association between an exposure and an outcome. Compared to traditional observational studies, MR can mitigate the impact of confounding factors. With the advancement of high‐throughput genomics and proteomics technologies in plasma, MR‐based approaches have facilitated the identification of potential therapeutic targets for various diseases.^15^, ^16^ However, to date, there have been few reports on studying allergic diseases by integrating GWAS and protein quantitative trait loci (pQTL) data.

**FIGURE 1:**
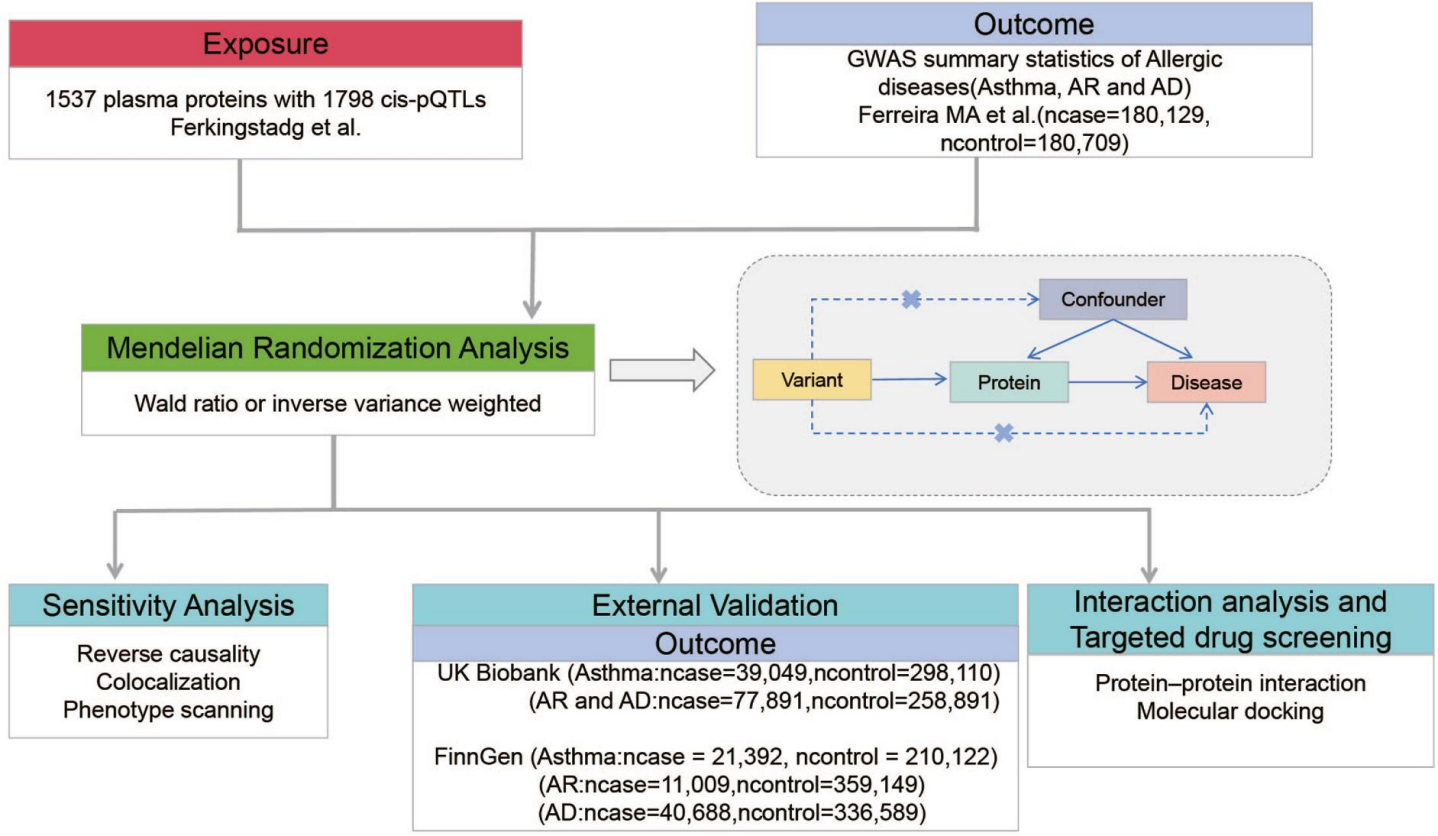
Study design: Identifying causal relationships between plasma proteins and allergic disease.

In this study, our aim was to identify plasma proteins as potential therapeutic targets for allergic diseases. The study design is illustrated in Figure 1. First, we employed GWAS data from Ferreira MA et al.^7^ and plasma pQTL data from Ferkingstad E et al.^17^ to identify potential plasma pathogenic proteins for allergic diseases using MR. Second, reverse causal association, Bayesian colocation analysis, and phenotype scanning were further employed to validate the findings. Third, we further validated our findings using two large external cohorts, namely the UK Biobank and FinnGen. Next, we constructed a protein‐protein interaction network to explore the mechanisms through which pathogenic proteins contribute to the occurrence of allergic diseases. Finally, based on the potential protein targets determined through MR and Bayesian Co‐location analysis, we conducted molecular docking to identify potential drugs for allergic diseases.

## 2 MATERIALS AND METHODS

### 2.1 Selection of plasma protein data

From the large‐scale protein quantitative trait loci (pQTL) study of 35,559 Icelanders, the summary statistical data related to 4907 circulating proteins were extracted.^17^ Proteomic analysis was performed using a multiplexed, modified aptamer‐based binding assay (SOMAscan version4). Protein levels underwent rank‐inverse normal transformation based on age and gender, and residuals were standardized through rank‐inverse normal transformation. The standardized values were treated as phenotypes in genome‐wide association analyses under the BOLT‐LMM linear mixed model. Detailed information on GWAS can be found in the original publication.^17^ This study included proteins with cis‐pQTLs obtained at the genome‐wide significant level (*p* < 5 × 10^−8^) in a two‐sample MR. Significant cis‐pQTLs was used for subsequent analysis.

### 2.2 GWAS summary data of outcome

The primary analysis utilized summary statistics from Ferreira MA et al.,^7^ encompassing 360,838 individuals of European ancestry (nCase = 180,129, nControl = 180,709). Further validation of the primary analysis results was conducted through external cohorts from FinnGen (R9 release, Asthma: nCase = 21,392, nControl = 210,122; AR: nCase = 11,009, nControl = 359,149; AD: nCase = 40,688, nControl = 336,589)^18^ and the UK Biobank (Asthma: nCase = 39,049, nControl = 298,110, AD and AR: nCase = 77,891, nControl = 258,891).^19^

## 3 STATISTICAL ANALYSIS

### 3.1 Mendelian randomization analysis and external validation

In this study, we utilized plasma proteins as the exposure, allergic diseases as the outcome, and employed the “TwoSampleMR” package (ver. 0.5.6) for MR analysis. If a specific protein has only one pQTL, the Wald ratio is employed. When two or more suitable pQTLs are available, inverse variance‐weighted MR (MR‐IVW) is applied, followed by heterogeneity analysis.^20^ The odds ratio of increasing the risk of allergic diseases is expressed as the standard deviation (SD) of the increase in plasma protein level.

In the primary analysis, we applied the Bonferroni correction to adjust for multiple tests. Then, we used the threshold *p*‐value of 0.05/1537 (*p* < 3.25 × 10^−5^) to screen the results, and the significant proteins were used for subsequent analysis. External validation of the initially identified crucial proteins was performed through MR analysis, where a *p*‐value <0.05 was considered significant. Finally, the estimated values of potential drug targets in FinnGen or UK Biobank were combined by the meta‐analysis of random effects.

### 3.2 Reversal analysis of causality

Following the same pQTLs selection criteria, we obtained 74 instrumental variables for allergic diseases. Subsequently, we performed bidirectional two‐sample univariable MR to assess potential reverse causation in our primary MR analysis. We employed five algorithms from the “TwoSampleMR” package (ver. 0.5.6), namely MR‐IVW, MR‐Egger, Weighted median, Simple mode, and Weighted mode, to estimate the effects. Additionally, Steiger filtering was conducted to ensure the directionality of the association between proteins and allergic diseases.^21^ The *p*‐value <0.05 is considered statistically significant.

### 3.3 Bayesian co‐localization analysis

We utilized the “coloc” package (ver. 5.1.0) for co‐localization analysis, where Bayesian co‐localization analysis is employed to assess the probability of two features sharing the same causal variable. Previous studies have indicated that Bayesian co‐localization provides posterior probabilities for five hypotheses, aiming to determine whether two traits share a single variant.^22^ In this study, we evaluated the posterior probabilities of Hypothesis 3 and Hypothesis 4 (PPH4). In hypothesis 3, the protein and allergic diseases are associated with the region through distinct variants, while in hypothesis 4, both the protein and allergic diseases are associated with the region through shared variants. The criterion for determining co‐localization between protein and allergic diseases was based on the Bayesian co‐localization analysis, specifically when the PPH4 posterior probability exceeded 0.8.^23^

### 3.4 Phenotype screening

We employed the “PhenoScanner” package (ver. 1.0)^24^ for phenotype screening, searching through previous GWAS to unveil associations of identified pQTLs with other traits. pQTL meeting the following criteria were considered pleiotropic: (i) the association is significant at a genome‐wide level (*p* < 5 × 10^−8^); (ii) the GWAS was conducted in populations of European ancestry; (iii) the SNP is associated with any known risk factors for allergic diseases, including metabolic traits, proteins, or clinical features.

### 3.5 Protein‐protein interaction network analysis

To investigate potential interactions among identified proteins, we utilize the STRING database (https://string‐db.org/) to construct a Protein‐Protein Interaction (PPI) network. Subsequently, we conduct a visual analysis of the Protein‐Protein Interaction (PPI) network based on STRING using Cytoscape software (ver. 3.9.1).

### 3.6 Targeted drug screening

After identifying potential drug targets based on MR and Bayesian co‐localization analyses, we utilized Autodock (Linux, ver. 4.2) for molecular docking to investigate the interaction of small molecular compounds with potential drug target genes. First, we obtained the catalog of small molecules interacting with potential drug target genes from the CTD database (https://ctdbase.org) and downloaded the structures of these small molecules from the PubChem database (https://pubchem.ncbi.nlm.nih.gov). Subsequently, we retrieved the biomacromolecule structure of the potential drug target gene from the UniProt database (https://www.uniprot.org). Finally, following the standard docking procedure, biological macromolecules and small molecular compounds were automatically docked, and those small molecular compounds demonstrating stable binding with biological macromolecules exhibited lower binding energy. Additionally, the results were visualized using PyMol (ver. 2.6, open source).

## 4 RESULTS

### 4.1 Protein‐wide mendelian randomization for screening causal proteins in allergic diseases

**FIGURE 2:**
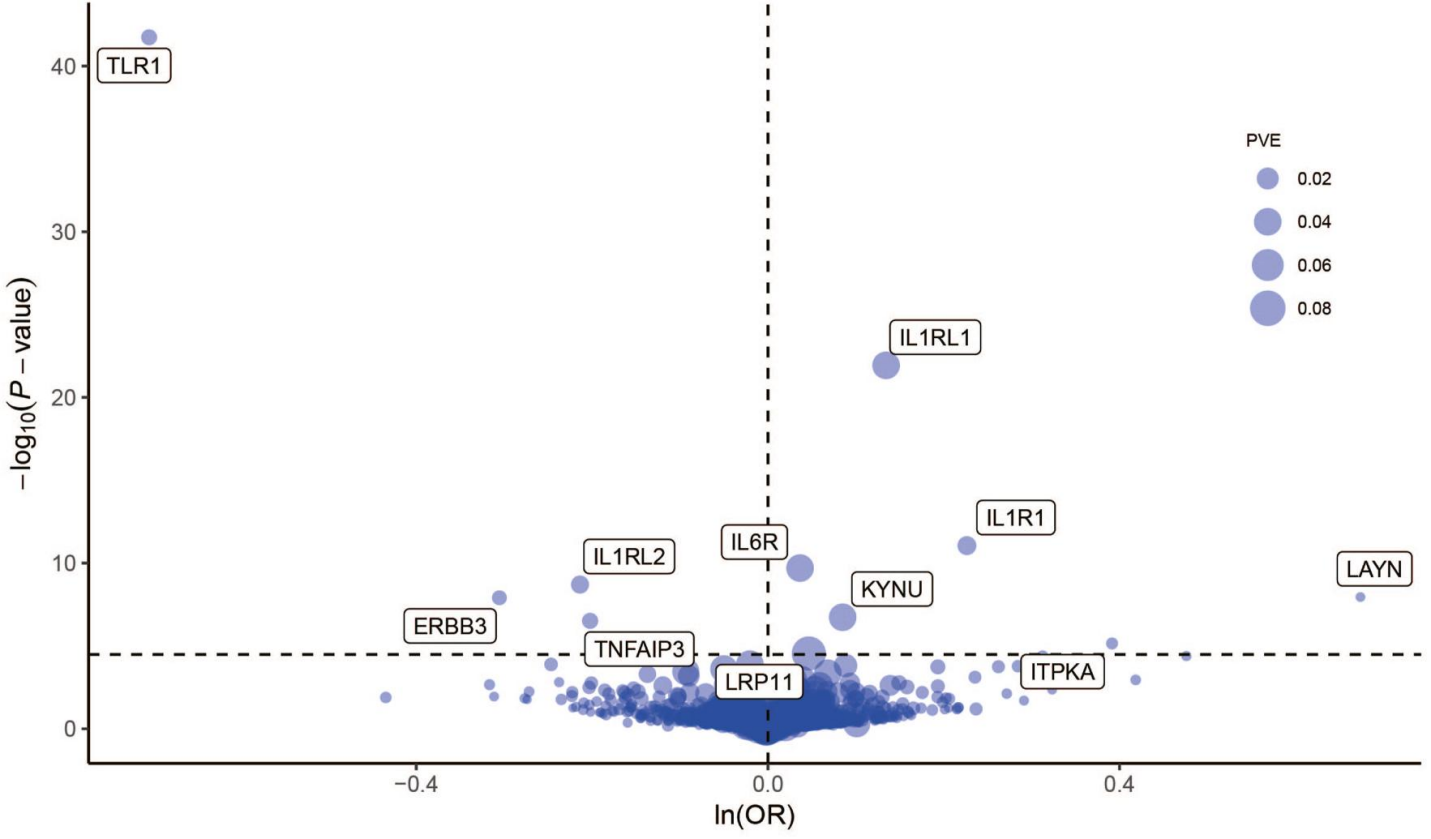
Mendelian Randomization (MR) results for plasma proteins and allergic disease risk. A volcano plot illustrates the MR results for 1537 plasma proteins concerning the risk of allergic diseases. The MR analysis for the association between plasma proteins and allergic disease risk employed either Wald ratio or inverse variance weighting. The odds ratio (OR) for increased risk of allergic diseases is presented as each standard deviation (SD) increase in plasma protein levels. The horizontal dashed line corresponds to a significance threshold of *p* = 3.25 × 10^‐5^ (0.05/1537). PVE, proportion of variance explained.

At Bonferroni significance (*p* < 3.25 × 10^−5^), the MR analysis revealed 11 significantly associated protein‐allergic diseases pairs (Table 1, Figure 2), namely, Interleukin‐6 receptor (IL6R), Interleukin‐1 receptor (IL1R1), TNF alpha induced protein 3 (TNFAIP3), Erb‐b2 receptor tyrosine kinase 3 (ERBB3), Inositol‐trisphosphate 3‐kinase A (ITPKA), Interleukin 1 receptor‐like 1 (IL1RL1), Toll‐like receptor 1 (TLR1), Kynureninase (KYNU), Layilin (LAYN), LDL receptor related protein 11 (LRP11), Interleukin 1 receptor like 2 (IL1RL2). Specifically, elevated levels of TNFAIP3, ERBB3, TLR1, and IL1RL2 proteins were associated with a reduced risk of allergic diseases, yielding corresponding odds ratios of 0.82 (0.76–0.88), 0.74 (0.66–0.82), 0.49 (0.45–0.55), and 0.81 (0.75–0.87), respectively. Conversely, increased levels of IL6R, IL1R1, ITPKA, IL1RL1, KYNU, LAYN, and LRP11 proteins were linked to an elevated risk of allergic diseases, with corresponding odds ratios of 1.04 (1.03–1.05), 1.25 (1.18–1.34), 1.48 (1.25–1.75), 1.14 (1.11–1.18), 1.09 (1.05–1.12), 1.96 (1.56–2.47), and 1.05 (1.03–1.07), respectively. Preliminary MR analysis showed no heterogeneity of pathogenic proteins of allergic diseases (Table S1).

**TABLE 1:** MR results: Plasma proteins were significantly linked with allergic diseases after Bonferroni correction.

Tisse	Protein	Uniprot ID	SNP^a^	Effect allele	OR(95% CI)^b^	P‐value	PVE	F statics	Author
Plasma	IL6R	P08887	rs12126142	A	1.04 (1.03, 1.05)	1.99e‐10	4.00%	1479.66	Ferkingstad
Plasma	IL1R1	P14778	rs7588201	C	1.25 (1.18, 1.34)	8.77e‐12	1.22%	437.93	Ferkingstad
Plasma	TNFAIP3	P21580	rs5029937	T	0.82 (0.76, 0.88)	3.05e‐07	0.69%	245.60	Ferkingstad
Plasma	ERBB3	P21860	rs773116	A	0.74 (0.66, 0.82)	1.23e‐08	0.50%	179.51	Ferkingstad
Plasma	ITPKA	P23677	rs316617	C	1.48 (1.25, 1.75)	7.06e‐06	0.21%	75.19	Ferkingstad
Plasma	IL1R1	P14778	rs7588201	C	1.25 (1.18, 1.34)	8.77e‐12	1.22%	437.93	Ferkingstad
Plasma	TNFAIP3	P21580	rs5029937	T	0.82 (0.76, 0.88)	3.05e‐07	0.69%	245.60	Ferkingstad
Plasma	ERBB3	P21860	rs773116	A	0.74 (0.66, 0.82)	1.23e‐08	0.50%	179.51	Ferkingstad
Plasma	ITPKA	P23677	rs316617	C	1.48 (1.25, 1.75)	7.06e‐06	0.21%	75.19	Ferkingstad
Plasma	IL1RL1	Q01638	rs13020553	G	1.14 (1.11, 1.18)	1.16e‐22	4.00%	1479.66	Ferkingstad
Plasma	TLR1	Q15399	rs5743618	A	0.49 (0.45, 0.55)	1.84e‐42	0.66%	236.66	Ferkingstad
Plasma	KYNU	Q16719	rs12477146	A	1.09 (1.05, 1.12)	1.85e‐07	4.00%	1479.66	Ferkingstad
Plasma	LAYN	Q6UX15	rs4938792	C	1.96 (1.56, 2.47)	1.12e‐08	0.11%	39.48	Ferkingstad
Plasma	LRP11	Q86VZ4	rs9479810	T	1.05 (1.03, 1.07)	2.85e‐05	7.21%	1479.66	Ferkingstad
Plasma	LRP11	Q86VZ4	rs9479094	T	1.05 (1.03, 1.07)	2.85e‐05	7.21%	1181.83	Ferkingstad
Plasma	IL1RL2	Q9HB29	rs3917265	T	0.81 (0.75, 0.87)	1.97e‐09	1.04%	374.08	Ferkingstad

^a^ All SNPs used were cis‐acting.

^b^ Odds ratios for increased risk of allergic diseases were expressed as per SD increase in plasma protein levels.

### 4.2 Sensitivity analysis for allergic diseases causal proteins

Following sensitivity analysis and MR analysis, we identified two proteins among the 11 significant ones as potential drug targets for allergic diseases, namely LAYN and TNFAIP3. Firstly, we conducted reverse MR analysis, and the results indicated that there were no causal effects of allergic diseases on the levels of 10 identified proteins, including IL6R, IL1R1, TNFAIP3, ERBB3, ITPKA, IL1RL1, TLR1, KYNU, LAYN and LRP11. Nonetheless, allergic diseases exert a causal effect on the protein level of IL1RL2, suggesting that IL1RL2 could serve as a potential biomarker for allergic diseases. Steiger filtering further ensured the directionality (Table 2 and Figure S1). Next, we performed Bayesian co‐localization analysis, and the results suggested that LAYN (coloc.abf‐PPH4 = 0.819) and TNFAIP3 (coloc.abf‐PPH4 = 0.930) share the same variant with allergic diseases (Table 2 and Figure S2). Finally, phenotypic scanning revealed some correlations, such as the correlation between IL6R and asthma, AD and eczema, the correlation between TNFAIP3 and some autoimmune diseases such as rheumatoid arthritis and Systemic lupus erythematosus (SLE), the correlation between ITPKA and the abundance of some immune cells, the correlation between ERBB3 and body mass index and thyroid dysfunction, the correlation between IL1RL1 and asthma and autoimmune diseases such as Crohn's disease and inflammatory bowel disease, and the correlation between TLR1 and allergic diseases such as asthma, hay fever and eczema (Table 2 and Table S2).

**TABLE 2:** Sensitivity analysis of causal proteins associated with allergic diseases.

Tissue	Protein	UniProt ID	SNP	Bidirectional MR (MR‐IVW)^c^	Steiger filtering	Co‐localization PPH4 (coloc.abf)	Previously reported
Plasma	IL6R	P08887	rs12126142	0.99 (0.92,1.06)	True(1.647 × 10^−261^)	0.026	Coronary artery disease^a^, ^b^, Asthma^a^, ^b^, Atopic dermatitis^a^, CRP^a^, ^b^
Plasma	IL1R1	P14778	rs7588201	0.99 (0.93,1.06)	True(1.644 × 10^−71^)	0.006	NA
Plasma	TNFAIP3	P21580	rs5029937	0.98 (0.93,1.03)	True(5.893 × 10^−41^)	0.930	Rheumatoid arthritis^b^, SLE^b^, Lupus erythematosus systemic^b^
Plasma	ERBB3	P21860	rs773116	0.97 (0.92,1.02)	True(1.814 × 10^−28^)	0.005	Body mass index^a^, Impedance of whole body^a^, Years of educational attainment^a^
Plasma	ITPKA	P23677	rs316617	1.02 (0.95,1.08)	True(4.356 × 1 0^−12^)	0.000	Sum basophil neutrophil counts^a^, Neutrophil percentage of white cells^a^
Plasma	IL1RL1	Q01638	rs13020553	0.97 (0.93,1.02)	True(2.701 × 10^−246^)	0.000	Asthma^a^, Crohns disease^b^, Inflammatory bowel disease^b^
Plasma	TLR1	Q15399	rs5743618	0.92 (0.82,1.02)	True(4.028 × 10^−26^)	0.006	Allergic disease^a^, Breast cancer^a^, *Helicobacter pylori* seroprevalence^b^
Plasma	KYNU	Q16719	rs12477146	1.03 (0.85,1.25)	True(1.060 × 10^−266^)	0.017	Lymphocyte count^a^, Platelet distribution width^a^, Kynureninase^a^
Plasma	LAYN	Q6UX15	rs4938792	1.00 (0.94,1.06)	True(1.843 × 10^−5^)	0.819	Self‐reported hypertension^a^
Plasma	LRP11	Q86VZ4	rs9479810	0.98 (0.94,1.02)	True(1.281 × 10^−273^)	0.002	NA
Plasma	LRP11	Q86VZ4	rs9479094	0.98 (0.94,1.02)	True(2.144 × 10^−216^)	0.002	NA
Plasma	IL1RL2	Q9HB29	rs3917265	0.92 (0.87,0.97)	True(4.644 × 10^−62^)	0.000	Interleukin‐1 receptor‐like 2^a^

Abbreviations: NA, not available; SLE, Systemic lupus erythematosus.

^a^ SNP is directly linked to phenotypic traits.

^b^ SNP with traits that are indirectly influenced through its proxy.

^c^ As the levels of plasma protein increased by one standard deviation, the risk of allergic diseases also increased.

### 4.3 External validation of potential drug targets for allergic diseases

**FIGURE 3:**
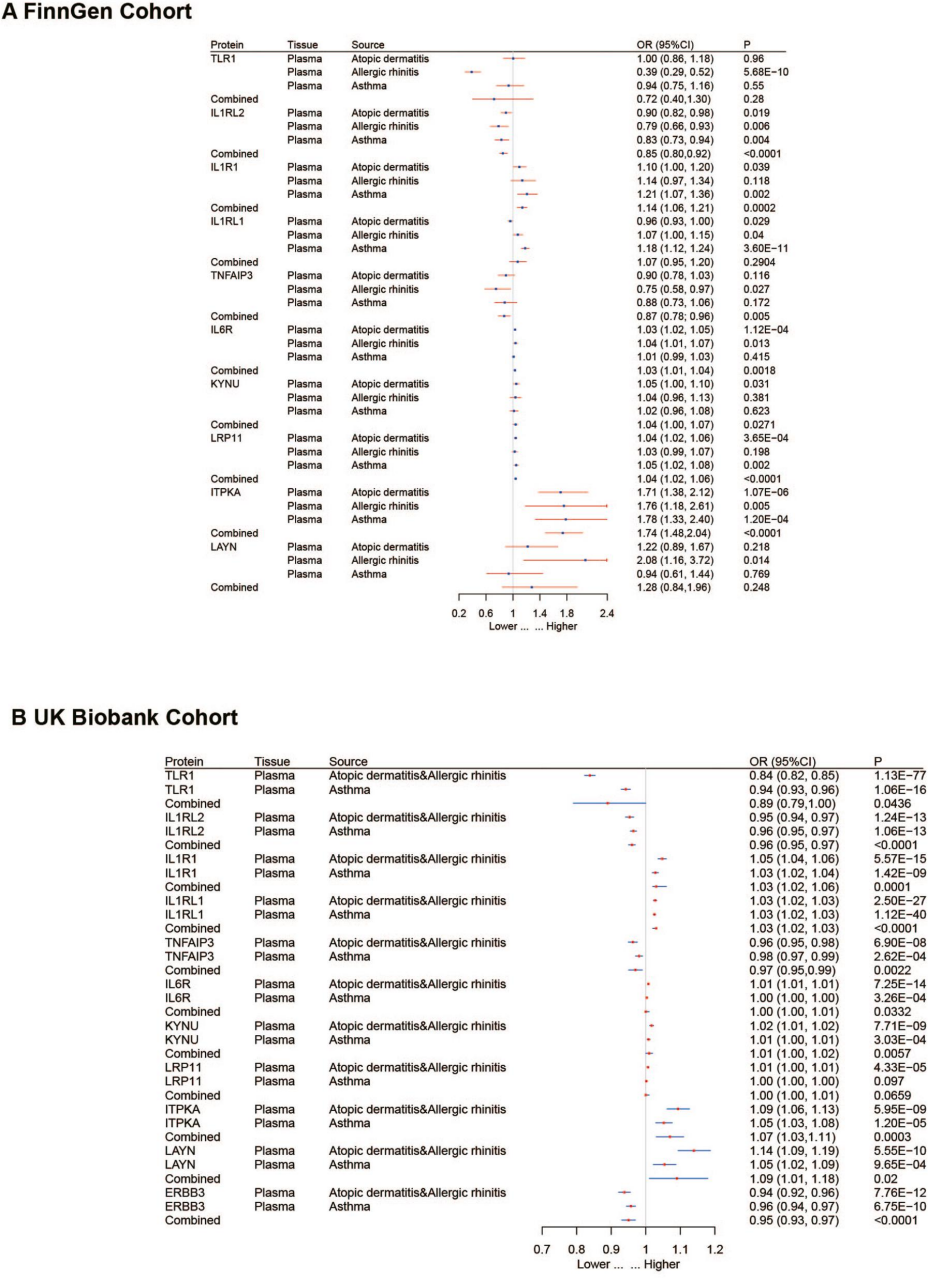
External validation of proteins potentially causally linked to allergic diseases using data from (A) FinnGen Cohort and (B) the UK Biobank Cohort. The increased risk of allergic diseases is represented by the odds ratio (OR) for each standard deviation (SD) increase in plasma protein levels.

To validate the accuracy of the preliminary MR analysis, we conducted validation in two external cohorts, FinnGen and UK Biobank. In the meta‐analysis of the FinnGen cohort, seven proteins showed significant associations, and the odds ratios (95% confidence intervals, CI) for allergic diseases per standard deviation increase in genetically predicted protein levels were as follows: TNFAIP3 0.87 (0.78–0.96), IL1RL2 0.85 (0.80–0.92), IL1R1 1.14 (1.06–1.21), IL6R 1.03 (1.01–1.04), KYNU 1.04 (1.00–1.07), LRP11 1.04 (1.02–1.06) and ITPKA 1.74 (1.48–2.04). In the meta‐analysis of the UK Biobank cohort, 10 proteins exhibited significant associations. The odds ratios (95% confidence intervals, CI) for allergic diseases per standard deviation increase in genetically predicted protein levels were as follows: TNFAIP3 0.97 (0.95–0.99), LAYN 1.09 (1.01–1.18), TLR1 0.89 (0.79–1.00), IL1RL2 0.96 (0.95–0.97), IL1R1 1.04 (1.02–1.06), IL1RL1 1.03 (1.02–1.03), IL6R 1.01 (1.00–1.01), KYNU 1.01 (1.00–1.02), ITPKA 1.07 (1.03–1.11) and ERBB3 0.95 (0.93–0.97) (Figure 3).

### 4.4 Protein‐protein interaction and potential target drug therapy

**FIGURE 4:**
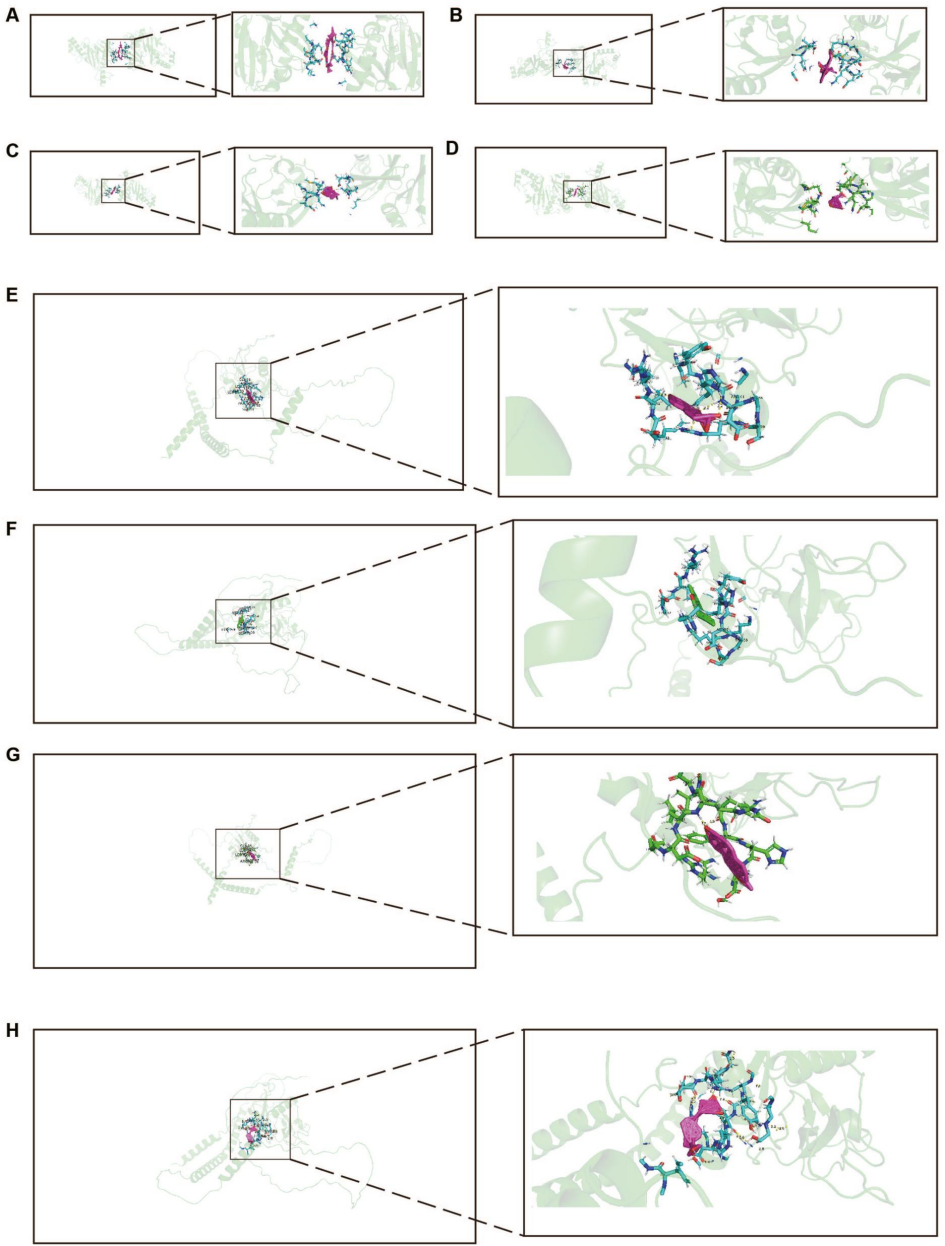
Targeted drug therapy (A) Cyclosporine (B) Vincristine (C) Withaferin A (D) Ursodeoxycholic acid (E) Dexamethasone (F) Dronabinol (G) Ethinyl estradiol (H) Calcitriol.

The protein‐protein interaction analysis revealed interactions among the identified potential causal proteins. Notably, interactions were observed between TNFAIP3 and IL1R1 as well as TLR1. Additionally, interactions were found among IL1RL1, IL1RL2, IL1R1, and IL6R. Moreover, interactions were noted between IL1R1 and TLR1 as well as IL6R. They mainly participate in regulating biological pathways such as inflammatory responses and immune reactions (Figure S3). Through MR and Bayesian colocalization analysis, we identified TNFAIP3 and LAYN as potential drug targets for allergic diseases. An increase in TNFAIP3 protein levels was negatively correlated with the risk of allergic diseases, whereas an increase in LAYN protein levels was positively correlated with the risk of allergic diseases. Therefore, we screened small molecular compounds from the CTD database that can increase TNFAIP3 expression and decrease LAYN expression, and performed molecular docking using Autodock. Four small molecular compounds can dock with TNFAIP3 increasing its expression. Four small molecular compounds can dock with LAYN decreasing its expression. The docking results of small molecular compounds with TNFAIP3 are shown in Figure 4A–D. The docking results of small molecular compounds with LAYN are shown in Figure 4E–H. Their simulated binding energies are −13.08 (kcal/mol), −9.39 (kcal/mol), −6.57 (kcal/mol), −6.44 (kcal/mol), −7.89(kcal/mol), −6.39(kcal/mol), −7.28 (kcal/mol) and −6.81 (kcal/mol), respectively (Table S3). In summary, we have identified four small molecular compounds that can upregulate TNFAIP3 expression and four that can downregulate LAYN expression, providing insights for targeted therapy of allergic diseases.

## 5 DISCUSSION

In this study, we used a two‐sample MR method to explore causal proteins related to allergic diseases. Ten potential drug targets for allergic diseases were identified, comprising TNFAIP3, LAYN, TLR1, IL1R1, IL1RL1, IL6R, KYNU, ITPKA, LRP11 and ERBB3. IL1RL2 was identified as a potential biomarker for allergic diseases. Bayesian co‐location analysis showed that TNFAIP3 and LAYN may have the same variants as allergic diseases. To validate the accuracy of the primary MR analysis results, we conducted external validation using the FinnGen and UK Biobank cohorts. The results demonstrated a significant association of TNFAIP3, IL1RL2, IL1R1, IL6R, KYNU, and ITPKA with allergic diseases in both cohorts, further supporting the reliability of the potential drug targets identified in this study.

The occurrence of diseases is a complex process primarily attributed to alterations in genes and proteins. Changes in genes lead to structural modifications in proteins, ultimately resulting in the onset of diseases. Consequently, alterations in proteins are closely linked to the occurrence of diseases. The human proteome serves as a key target for disease therapy. Therefore, to identify novel drug targets for allergic diseases, we employed a comprehensive approach integrating MR and Bayesian colocalization to assess the causal proteins associated with allergic diseases.^25^ First of all, in order to eliminate the influence of reverse causality, we conducted a bidirectional MR analysis. Bidirectional MR analysis did not reveal any causal effects of allergic diseases on the levels of 10 identified proteins. Further validation of the directionality of causation was performed through Steiger filtering. Second, to mitigate the impact of horizontal pleiotropy, we exclusively utilized cis‐pQTLs as instrumental variables. This choice was made because these loci directly influence the transcription and translation processes of the associated genes.^26^ Next, we employed Bayesian colocalization analysis to eliminate biases caused by linkage disequilibrium. With a critical threshold of 0.8 for the posterior probability 4, two proteins identified through MR analysis may share the same variant with allergic diseases.^27^ Finally, through phenotypic screening, we discovered that the protein targets identified by MR were associated with various traits. However, these associations are insufficient to fully elucidate the relationship between proteins and allergic diseases. This study suggests that TNFAIP3 and LAYN may serve as potential drug targets for allergic diseases.

Allergic diseases (asthma, AR, and AD) often coexist in the same individual,^28^ partially due to shared genetic origins.^29^, ^30^, ^31^ While many targets for allergic diseases (asthma, AR, and AD) have been discovered in recent years, developing new drugs or repurposing existing ones for the treatment of these diseases remains challenging. Ferreira MA et al.^7^ identified drug targets for a range of allergic diseases based on GWAS and eQTL analyses, while our study utilized pQTLs to identify potential drug targets for allergic diseases. Therefore, our study is an important addition to the research by Ferreira MA et al. in identifying drug targets for allergic diseases, which is crucial for the development of market‐approved drugs.

TNFAIP3 (Tumor Necrosis Factor Alpha‐Inducible Protein 3) is a gene that encodes the TNFAIP3 (A20) protein, serving as a critical anti‐inflammatory regulatory factor.^32^ It plays a significant role in anti‐inflammatory responses,^33^ autoimmune diseases,^34^, ^35^ and the development of tumors.^36^, ^37^ Specifically, TNFAIP3 regulates the anti‐inflammatory process of the immune system by inhibiting the NF‐*κ*B signaling pathway and reducing the production of inflammatory factors.^38^ Abnormal expression or mutation of TNFAIP3 is closely related to the pathogenesis of autoimmune diseases.^39^ The relationship between TNFAIP3 and tumors is complex and multifaceted. On one hand, TNFAIP3 can reduce the progression of inflammation‐related tumors. On the other hand, the abnormal expression of TNFAIP3 may promote the development of tumor.^40^ However, it remains unclear whether TNFAIP3 plays a role in allergic diseases. Based on MR and Bayesian co‐location analysis, we identified TNFAIP3 as a potential drug target for allergic diseases. Besides, through phenotype screening, we identified an association between TNFAIP3 (rs5029937) and various autoimmune diseases, such as rheumatoid arthritis and SLE, consistent with previous research. Finally, our protein interaction network analysis shows that there are interactions between TNFAIP3 and TLR1 and/or IL1R1. This suggests that TNFAIP3 may exert its associated functions through interactions with TLR1 and/or IL1R1.

TLR1 Toll‐like Receptor 1 is a member of the Toll‐like receptor family and plays a crucial role in the immune system.^41^ Previous research has demonstrated that TLR1 plays a crucial role in the occurrence and development of various diseases, including infectious diseases,^42^ autoimmune diseases,^43^ and cancer.^44^ Specifically, TLR1 activates downstream signaling pathways, primarily involving NF‐*κ*B, MAPK, and IRF, through interactions with co‐receptors and adapter.^45^ The activation of these signaling pathways promotes the inflammatory response and the activation of immune cell, which plays a vital role in anti‐infection^46^ and tumor immune responses.^44^ However, it is not clear whether TLR1 plays a role in allergic diseases and the possible mechanisms involved. In this study, through phenotype screening, we found that TLR1 (rs5743618) is associated with allergic diseases, breast cancer, and the positivity rate of *Helicobacter pylori* in serum. Therefore, we think that TLR1 may be expected to be a therapeutic target for allergic diseases.

IL1R1 is a membrane‐bound protein, which can also be cleaved into a soluble circulating form by matrix metalloproteinases. Both the membrane‐bound and soluble forms of IL1R1 have biological activity, which regulates the inflammatory response by activating and antagonizing cytokine activity.^47^, ^48^ Studies have demonstrated the association of IL1R1 with autoimmune diseases, including rheumatoid arthritis,^49^ as well as cancers such as breast cancer^50^ and colorectal cancer,^51^ along with inflammatory diseases like inflammatory bowel disease.^52^ First of all, our research shows that the expression level of IL1R1 protein is related to the occurrence of allergic diseases. Next, we have been further verified in two large independent external cohorts, providing further evidence for IL1R1 as a potential target for allergic diseases. Finally, protein‐protein interaction network analysis reveals interactions between IL1R1 and TLR1, TNFAIP3, IL6R, and IL1RL1. In conclusion, IL1R1 could be a potential therapeutic target for allergic diseases.

LAYN, a widely expressed integral membrane hyaluronan receptor, encodes Layilin, a membrane glycoprotein featuring the structural domain of C‐type lectin.^53^ Prior studies have indicated that LAYN is associated with cellular senescence, expressed in specific cells and organs, and plays a crucial role in regulating motility, cell adhesion, and migration.^54^, ^55^ Furthermore, LAYN plays a role in cancer development and enhances anti‐tumor immunity by promoting the activation of integrins.^56^ In this study, we identified LAYN as a potential drug target for allergic diseases through MR and Bayesian co‐location analysis.

Our research shows that TNFAIP3 and LAYN are potential drug targets for allergic diseases. The elevation of TNFAIP3 protein levels is associated with a reduced risk of allergic diseases, whereas the increase in LAYN protein levels is correlated with an elevated risk of allergic diseases. Therefore, we screened four small molecular compounds from the CTD database that can downregulate the expression levels of LAYN, including dexamethasone, dronabinol, ethinylestradiol, and calcitriol. Their simulated binding energies are −7.89(kcal/mol), −6.39(kcal/mol), −7.28 (kcal/mol), −6.81 (kcal/mol). Additionally, four small molecular compounds that elevate TNFAIP3 expression levels were identified, including cyclosporine, vincristine, Withaferin A (WA), and Ursodeoxycholic Acid (UDCA). Their simulated binding energies are −13.08 (kcal/mol), −9.39 (kcal/mol), −6.57 (kcal/mol), −6.44 (kcal/mol). Molecular docking studies show that these small molecules can effectively and stably bind TNFAIP3 and/or LAYN.^57^ Dexamethasone is a synthetic corticosteroid hormone that has been extensively utilized in the treatment of various conditions, including autoimmune diseases, allergies, eye disorders, cancer, and more recently COVID‐19.^58^ Dronabinol is a synthetic cannabinoid medication, with its active ingredient being Δ9‐tetrahydrocannabinol (THC). It has been approved and widely utilized for alleviating nausea and vomiting in chemotherapy patients.^59^ Ethinyl Estradiol is a potent synthetic estrogen widely employed in oral contraceptives and also utilized in the treatment of breast cancer and prostate cancer.^60^ Calcitriol, an active form of vitamin D commonly referred to as vitamin D3, plays a significant role in maintaining bone health and in the prevention^61^ and treatment of cancer.^62^ Cyclosporine is an immunosuppressive agent that has demonstrated effective therapeutic outcomes in the treatment of conditions such as psoriasis, hallopeau continuous limb dermatitis, necrotizing pyoderma, lichen planus, chronic lichenoid pityriasis, Behçet's disease, and alopecia areata.^63^ Vincristine belongs to the ergot alkaloid family and is typically utilized in the treatment of various types of cancer, including leukemia^64^ and lymphoma.^65^ WA is a pivotal drug with diverse mechanisms and extensive pharmacological characteristics. It possesses anticancer, anti‐inflammatory, anti‐herpes, anti‐fibrosis, anti‐platelet, fibrinolytic, immunosuppressive, anti‐pigmentation, anti‐parasite, and healing potential.^66^ UDCA is a natural bile acid used clinically for the treatment of conditions associated with bile stasis.^67^

Our research has some limitations. First of all, although we have studied the correlation between plasma protein level and the risk of allergic diseases (asthma, AR and AD) through genetic method, more clinical research is needed to further verify our research. In future research, it is imperative to incorporate additional clinical research to assess the impact of plasma proteins on the susceptibility to allergic diseases. Secondly, our study was conducted in populations of European descent to minimize population stratification biases. However, due to the variability in ethnic backgrounds, our study may not be generalizable to other ancestral populations.^15^, ^68^, ^69^ When we have more GWAS data on allergic diseases from additional ancestral backgrounds, we plan to conduct supplementary studies. This will enable us to further explore our research findings and facilitate the translation of our findings into clinical applications. Thirdly, we employed stringent selection criteria, utilizing a strict *p*‐value threshold (*p* < 5 × 10^−8^) and cis‐acting pQTLs as instrumental variables, which also constrained the applicability of some analyses, including alternative MR algorithms, heterogeneity testing, and pleiotropy testing. However, through the calculation of F‐statistics, we observed that the F‐statistics for the instrumental variables we screened were all greater than 10. F‐statistics greater than 10 exclude the influence of weak instrumental variables. Fourth, asthma is a heterogeneous disease with several subtypes (allergic and non‐allergic) and has a variable genetic background.^70^ In this study, we employed a genome‐wide association study (GWAS) to identify proteins associated with the risk of allergic diseases (asthma, AR and AD). However, due to the heterogeneity and variable genetic background of allergic diseases, it is necessary to combine various methods (family research, in‐depth sequencing, epigenetics, population stratification, functional research and clinical research) to deeply understand the pathogenesis, subtypes and individual differences. Finally, we employed Protein‐Protein Interaction (PPI) analysis to examine the interactions between proteins and utilized molecular docking to identify potential drug candidates. However, the findings from the PPI analysis and molecular docking are preliminary, requiring further validation in future research.

## 6 CONCLUSION

In summary, our comprehensive analysis indicates a causal relationship between genetically determined circulating levels of TNFAIP3, LAYN, IL6R, TLR1, IL1R1, IL1RL2, ERBB3, LRP11, KYNU, ITPKA, and IL1RL1 and the risk of allergic diseases. The identified proteins may serve as attractive drug targets/biomarker for allergic diseases, particularly TNFAIP3 and LAYN. Further research is warranted to explore the roles of these candidate proteins in allergic diseases.

## AUTHOR CONTRIBUTIONS

Hui Wang: Concept, Manuscript writing and design; Wenru Tang, Juhua Dan and Miaomiao Sheng: Administrative support; Qi Qi, Y Tang, Yuheng Tang and Samina Gul: Collection of data; Hui Wang and Jianyu Pang: Data analysis; All authors: Approved the final manuscript.

## CONFLICT OF INTEREST STATEMENT

The authors declare that they have no competing interests in this section.

## CONSENT TO PARTICIPATE

Not applicable.

## CONSENT FOR PUBLICATION

Not applicable.

## Supporting information

Figure S1

Figure S2

Figure S3

Table S1

Table S2

Table S3

## Data Availability

This paper analyzes existing and publicly available data, with all GWAS sources duly cited. The original code is not provided in this article. For additional inquiries, please contact the corresponding author directly.
