# Supplementary material for: Identification of potential drug targets for allergic diseases from a genetic perspective: A mendelian randomization study
Source: Clin Transl Allergy. 2024 Apr 4;14(4):e12350. doi: 10.1002/clt2.12350 (PMC10994001; doi:10.1002/clt2.12350)
Supplement: Supplementary file 1 — Figure S1 [file CLT2-14-e12350-s002.pdf]

Protein	Method	OR (95%CI)	P
IL1R1	Inverse variance weighted	0.99 (0.92, 1.06)	0.766
	MR Egger	1.20 (0.98, 1.46)	0.086
	Weighted median	0.97 (0.90, 1.04)	0.393
	Simple mode	1.00 (0.85, 1.17)	0.971
	Weighted mode	0.96 (0.83, 1.10)	0.541
IL1RL1	Inverse variance weighted	0.97 (0.93, 1.02)	0.273
	MR Egger	1.00 (0.87, 1.13)	0.951
	Weighted median	0.97 (0.91, 1.04)	0.408
	Simple mode	0.96 (0.84, 1.10)	0.593
	Weighted mode	0.96 (0.85, 1.10)	0.570
IL1RL2	Inverse variance weighted	0.92 (0.87, 0.97)	0.003
	MR Egger	0.71 (0.61, 0.82)	1.37e−05
	Weighted median	0.93 (0.86, 0.99)	0.028
	Simple mode	0.95 (0.84, 1.08)	0.433
	Weighted mode	0.94 (0.84, 1.04)	0.224
IL6R	Inverse variance weighted	0.99 (0.93, 1.06)	0.844
	MR Egger	0.99 (0.83, 1.18)	0.925
	Weighted median	0.98 (0.92, 1.05)	0.600
	Simple mode	0.97 (0.85, 1.10)	0.623
	Weighted mode	0.96 (0.86, 1.07)	0.419
ITPKA	Inverse variance weighted	1.02 (0.95, 1.08)	0.635
	MR Egger	0.89 (0.75, 1.07)	0.212
	Weighted median	1.00 (0.93, 1.07)	0.933
	Simple mode	1.03 (0.89, 1.18)	0.706
	Weighted mode	1.04 (0.92, 1.17)	0.555
KYNU	Inverse variance weighted	1.03 (0.85, 1.25)	0.763
	MR Egger	0.81 (0.47, 1.40)	0.456
	Weighted median	0.94 (0.88, 1.01)	0.092
	Simple mode	0.94 (0.82, 1.09)	0.433
	Weighted mode	0.92 (0.82, 1.02)	0.118
LAYN	Inverse variance weighted	1.00 (0.94, 1.06)	0.938
	MR Egger	0.83 (0.71, 0.98)	0.027
	Weighted median	0.96 (0.89, 1.03)	0.244
	Simple mode	0.96 (0.84, 1.11)	0.627
	Weighted mode	0.91 (0.80, 1.04)	0.157
LRP11	Inverse variance weighted	0.98 (0.94, 1.02)	0.370
	**MR Egger**	**0.86 (0.77, 0.97)**	**0.014**
	Weighted median	0.98 (0.92, 1.04)	0.509
	Simple mode	0.98 (0.83, 1.16)	0.802
	Weighted mode	0.96 (0.84, 1.10)	0.584
TLR1	Inverse variance weighted	0.92 (0.82, 1.02)	0.102
	MR Egger	0.63 (0.48, 0.84)	0.002
	Weighted median	1.01 (0.95, 1.08)	0.743
	Simple mode	1.02 (0.88, 1.17)	0.833
	Weighted mode	1.03 (0.90, 1.17)	0.711
TNFAIP3	Inverse variance weighted	0.98 (0.93, 1.03)	0.354
	MR Egger	0.92 (0.80, 1.06)	0.241
	Weighted median	0.98 (0.91, 1.06)	0.625
	Simple mode	0.96 (0.83, 1.12)	0.623
	Weighted mode	0.99 (0.88, 1.10)	0.805
ERBB3	Inverse variance weighted	0.97 (0.92, 1.02)	0.190
	MR Egger	0.86 (0.75, 1.00)	0.053
	Weighted median	0.94 (0.87, 1.00)	0.059
	Simple mode	0.92 (0.78, 1.08)	0.300
	Weighted mode	0.91 (0.81, 1.03)	0.148

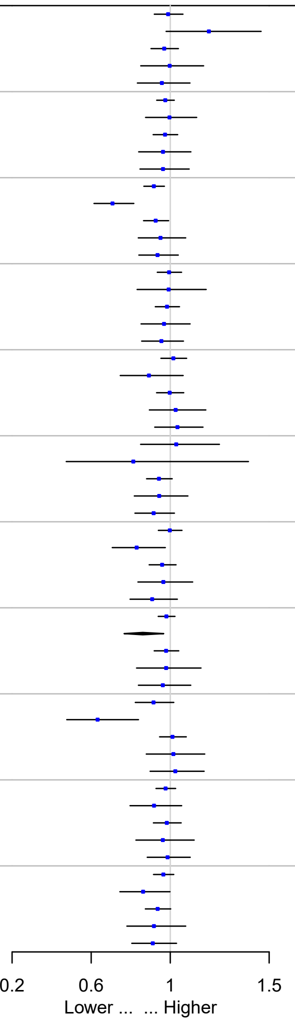

Supplementary Fig.1 Bidirectional Mendelian Randomization (MR) analysis of the levels of eleven potential causal proteins in allergic diseases. The odds ratio (OR) for increased risk of allergic diseases is presented as each standard deviation (SD) increase in plasma protein levels.
