# Supplementary material for: Identification of potential drug targets for allergic diseases from a genetic perspective: A mendelian randomization study
Source: Clin Transl Allergy. 2024 Apr 4;14(4):e12350. doi: 10.1002/clt2.12350 (PMC10994001; doi:10.1002/clt2.12350)
Supplement: Supplementary file 2 — Figure S2 [file CLT2-14-e12350-s004.pdf]

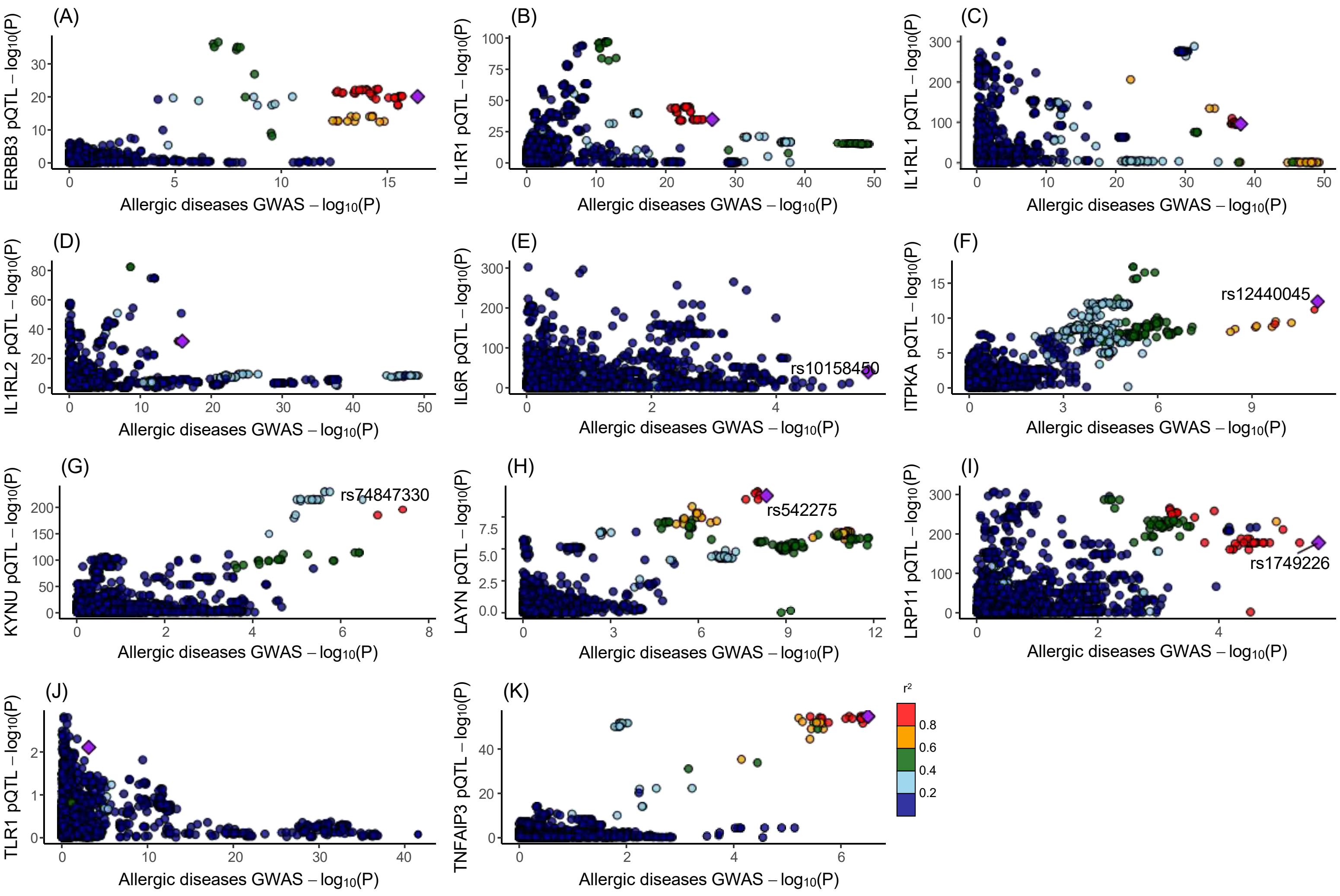

Supplementary Fig.2 Bayesian colocalization analysis of eleven potential causal proteins and allergic diseases. (A) ERBB3 (B) IL1R1 (C) IL1RL1 (D) IL1RL2 (E) IL6R (F) ITPKA (G) KYNU (H) LAYN (I) LRP11 (J) TLR1 (K) TNFAIP3
