# Supplementary material for: Identification of potential drug targets for allergic diseases from a genetic perspective: A mendelian randomization study
Source: Clin Transl Allergy. 2024 Apr 4;14(4):e12350. doi: 10.1002/clt2.12350 (PMC10994001; doi:10.1002/clt2.12350)
Supplement: Supplementary file 3 — Figure S3 [file CLT2-14-e12350-s006.pdf]

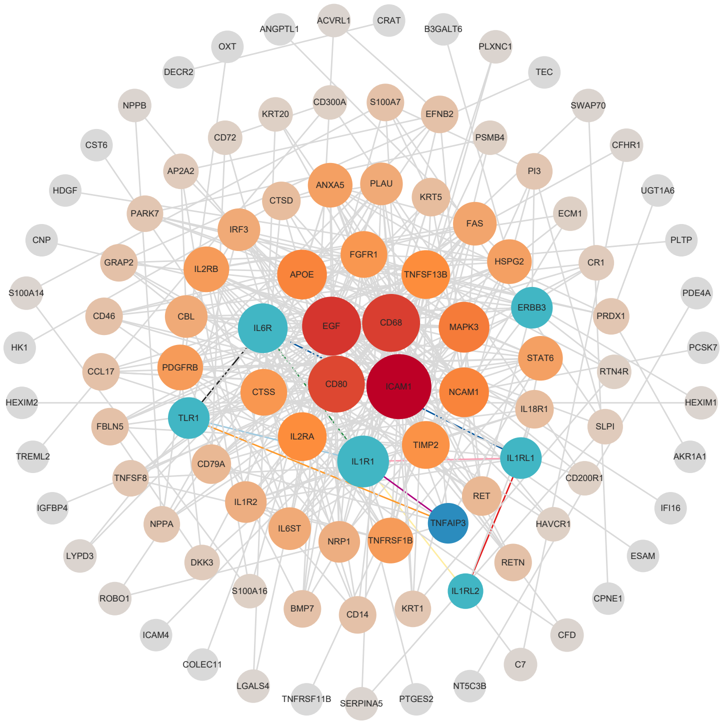

Supplementary Fig.3 Protein-protein interaction network analysis suggests potential mechanisms by which causal proteins ($P < 0.05$) contribute to the occurrence of allergic diseases.
