# Supplementary material for: Identification of potential drug targets for allergic diseases from a genetic perspective: A mendelian randomization study
Source: Clin Transl Allergy. 2024 Apr 4;14(4):e12350. doi: 10.1002/clt2.12350 (PMC10994001; doi:10.1002/clt2.12350)
Supplement: Supplementary file 4 — Table S1 [file CLT2-14-e12350-s001.pdf]

Tissue	Protein	method	Q statistics	Q_df	P-value (heterogeneity)
Plasma	C1QC	MR Egger	1.706923235	1	0.191385052
Plasma	GLTPD2	MR Egger	0.503403213	1	0.478008585
Plasma	PLXNC1	MR Egger	3.716789561	2	0.15592272
Plasma	CLSTN1	MR Egger	1.676315778	1	0.195414719
Plasma	KNG1	MR Egger	0.383068165	1	0.535965763
Plasma	NCAM1	MR Egger	1.337690853	1	0.247441602
Plasma	VEGFA	MR Egger	6.899205512	1	0.008623406
Plasma	CBLN1	MR Egger	2.870225852	1	0.090232207
Plasma	CNTFR	MR Egger	0.42767282	1	0.513133075
Plasma	THBS2	MR Egger	1.497534922	1	0.221051044
Plasma	CXCL12	MR Egger	4.855323118	3	0.182702853
Plasma	LUM	MR Egger	0.038893215	1	0.843660367
Plasma	FAM3B	MR Egger	0.738502278	1	0.390141075
Plasma	CHIT1	MR Egger	3.711552087	1	0.054037121
Plasma	TMEM132D	MR Egger	2.48940358	1	0.114615151
Plasma	FJX1	MR Egger	0.04709148	1	0.828203893
Plasma	LILRA2	MR Egger	3.125198942	1	0.077090462
Plasma	ADAMTSL1	MR Egger	0.344015816	1	0.557520338
Plasma	SEZ6L	MR Egger	0.162939686	1	0.686464315
Plasma	CLSTN2	MR Egger	0.885116143	1	0.346804228
Plasma	CPQ	MR Egger	0.000292065	1	0.986364892

Supplementary Table.1 Heterogeneity analysis of proteins with three or more instrumental variables.
