# Supplementary material for: Identification of potential drug targets for allergic diseases from a genetic perspective: A mendelian randomization study
Source: Clin Transl Allergy. 2024 Apr 4;14(4):e12350. doi: 10.1002/clt2.12350 (PMC10994001; doi:10.1002/clt2.12350)
Supplement: Supplementary file 5 — Table S2 [file CLT2-14-e12350-s003.pdf]

Tissue	Protein	UniProt	SNP	trait	catalog	Effect allele	proxy_SNP	r2	Proxy effect allele	beta	se	pval	ancestry	pmid
Plasma	**IL6R**	P08887	rs12126142	Corory artery disease	**GWAS**	**A**	rs4129267	1	A	-0.05046	0.008934	1.66E-08	Mixed	23202125
Plasma	**IL6R**	P08887	rs12126142	C reactive protein	**GWAS**	**A**	rs4129267	1	A			8.80E-12	European	23505291
Plasma	**IL6R**	P08887	rs12126142	Corory artery disease	**GWAS**	**A**	rs4129267	1	A			1.66E-08	Mixed	23202125
Plasma	**IL6R**	P08887	rs12126142	Interleukin 6 IL 6 levels	**GWAS**	**A**	rs4129267	1	A			2.36E-08	European	22291609
Plasma	**IL6R**	P08887	rs12126142	Plasma C reactive protein female	**GWAS**	**A**	rs4129267	1	A			1.97E-08	European	18439548
Plasma	**IL6R**	P08887	rs12126142	Plasma fibrinogen females	**GWAS**	**A**	rs4129267	1	A			1.84E-11	European	20031577
Plasma	**IL6R**	P08887	rs12126142	Soluble IL6R sIL6R	**GWAS**	**A**	rs4129267	1	A			2.49E-76	European	18464913
Plasma	**IL6R**	P08887	rs12126142	Corory artery disease	**GWAS**	**A**	rs4129267	1	A	-0.0506	0.0075	1.26E-11	Mixed	28530674
Plasma	**IL6R**	P08887	rs12126142	Asthma	**GWAS**	**A**	rs4129267	1	A	0.08618	0.01536	2.00E-08	European	21907864
Plasma	**IL6R**	P08887	rs12126142	C reactive protein levels	**GWAS**	**A**	rs4129267	1	A	-0.079	0.005402	2.00E-48	European	21300955
Plasma	**IL6R**	P08887	rs12126142	Fibrinogen	**GWAS**	**A**	rs4129267	1	A	0.011	0.001023	6.00E-27	Mixed	23969696
Plasma	**IL6R**	P08887	rs12126142	Protein quantitative trait loci	**GWAS**	**A**	rs4129267	1	A			2.00E-57	European	18464913
Plasma	**IL6R**	P08887	rs12126142	Asthma	**GWAS**	**A**	rs4129267	1	A			2.00E-08	European	21907864
Plasma	**IL6R**	P08887	rs12126142	C reactive protein	**GWAS**	**A**	rs4129267	1	A			2.00E-48	European	21300955
Plasma	**IL6R**	P08887	rs12126142	Receptors interleukin 6	**GWAS**	**A**	rs4129267	1	A			2.00E-57	European	18464913
Plasma	**IL6R**	P08887	rs12126142	Corory artery disease	**GWAS**	**A**	rs4129267	1	A	-0.0397	0.0052	2.07E-14	Mixed	29212778
Plasma	**IL6R**	P08887	rs12126142	Monocyte percentage of white cells	**GWAS**	**A**	rs2228145	1	A	0.02208	0.003603	8.98E-10	European	27863252

Plasma	**IL6R**	P08887	rs12126142	Circulating C reactive protein	**GWAS**	**A**	rs2228145	1	A			2.00E-08	European	23505291
Plasma	**IL6R**	P08887	rs12126142	Circulating interleukin 6 IL 6	**GWAS**	**A**	rs2228145	1	A			2.36E-08	European	23505291
Plasma	**IL6R**	P08887	rs12126142	Circulating soluble interleukin 6 sIL 6	**GWAS**	**A**	rs2228145	1	A			2.00E-57	European	23505291
Plasma	**IL6R**	P08887	rs12126142	Fibrinogen	**GWAS**	**A**	rs2228145	1	A			1.80E-11	European	23505291
Plasma	**IL6R**	P08887	rs12126142	Interleukin 6 IL 6 levels	**GWAS**	**A**	rs2228145	1	A			1.02E-21	European	23505291
Plasma	**IL6R**	P08887	rs12126142	Rheumatoid arthritis	**GWAS**	**A**	rs2228145	1	A			1.30E-08	European	23143596
Plasma	**IL6R**	P08887	rs12126142	Atopic dermatitis	**GWAS**	**A**	rs2228145	1	A	0.07696	0.01237	5.00E-10	Mixed	26482879
Plasma	**IL6R**	P08887	rs12126142	C reactive protein	**GWAS**	**A**	rs2228145	1	A			2.00E-08	European	18439548
Plasma	**IL6R**	P08887	rs12126142	Fibrinogen	**GWAS**	**A**	rs2228145	1	A	5.3	0.7903	2.00E-11	European	20031577
Plasma	**IL6R**	P08887	rs12126142	Fibrinogen levels	**GWAS**	**A**	rs2228145	1	A			9.00E-29	European	28107422
Plasma	**IL6R**	P08887	rs12126142	C-reactive protein	**GWAS**	**A**	rs2228145	1	A	-0.11	0.02	1.96E-10	European	28887542
Plasma	**IL6R**	P08887	rs12126142	Fibrinogen	**GWAS**	**A**	rs2228145	1	A			2.00E-11	European	20031577
Plasma	**IL6R**	P08887	rs12126142	Interleukin 6R	**GWAS**	**A**	rs12730935	1	A			4.87E-60	Mixed	23696881
Plasma	**IL6R**	P08887	rs12126142	Fibrinogen levels	**GWAS**	**A**	rs61812598	1	A			3.00E-36	European	26561523
Plasma	**IL6R**	P08887	rs12126142	Self-reported eczema or dermatitis	**GWAS**	**A**	rs61812598	1	A	0.00228	0.000393	6.51E-09	European	UKBB
Plasma	**IL6R**	P08887	rs12126142	P08887 protein abundance levels	**pQTL**	**A**						1.86E-08	European	22595970
Plasma	**IL6R**	P08887	rs12126142	Interleukin-6 receptor subunit alpha	**pQTL**	**A**	rs4129267	1	A	0.8092	0.0212	2.19E-265	European	28369058
Plasma	**IL6R**	P08887	rs12126142	P08887 protein abundance levels	**pQTL**	**A**	rs4129267	1	A			1.86E-08	European	22595970

Plasma	**IL6R**	P08887	rs12126142	Interleukin-6 receptor subunit alpha	**pQTL**	**A**	rs4129267	1	A	1.208	0.02477	1.58E-265	European	28240269
Plasma	**IL6R**	P08887	rs12126142	CRP	**pQTL**	**A**	rs4129267	1	A			2.00E-48	European	21300955
Plasma	**IL6R**	P08887	rs12126142	D-dimer	**pQTL**	**A**	rs4129267	1	A			6.00E-27	Mixed	23969696
Plasma	**IL6R**	P08887	rs12126142	IL-6 sRa	**pQTL**	**A**	rs4129267	1	A			1.56E-58	European	23894628
Plasma	**IL6R**	P08887	rs12126142	IL-6 sRa	**pQTL**	**A**	rs4129267	1	A			4.39E-58	European	25147954
Plasma	**IL6R**	P08887	rs12126142	P08887 protein abundance levels	**pQTL**	**A**	rs2228145	1	A			1.86E-08	European	22595970
Plasma	**IL6R**	P08887	rs12126142	P08887 protein abundance levels	**pQTL**	**A**	rs61812598	1	A			1.86E-08	European	22595970
Plasma	**IL6R**	P08887	rs12126142	D-dimer	**pQTL**	**A**	rs61812598	1	A			3.00E-36	European	26561523
Plasma	**TNFAIP3**	P21580	rs5029937	Rheumatoid arthritis	**GWAS**	**T**						3.16E-08	European	20453842
Plasma	**TNFAIP3**	P21580	rs5029937	Systemic lupus erythematosus	**GWAS**	**T**				0.5365	0.07425	5.00E-13	European	19838195
Plasma	**TNFAIP3**	P21580	rs5029937	Systemic lupus erythematosus or rheumatoid arthritis	**GWAS**	**T**	rs5029924	1	T			2.00E-19	European	27193031
Plasma	**TNFAIP3**	P21580	rs5029937	Rheumatoid arthritis	**GWAS**	**T**	rs5029939	1	G			1.21E-08	European	17554300
Plasma	**TNFAIP3**	P21580	rs5029937	Systemic lupus erythematosus SLE	**GWAS**	**T**	rs5029939	1	G			2.86E-12	European	19165918
Plasma	**TNFAIP3**	P21580	rs5029937	Systemic lupus erythematosus	**GWAS**	**T**	rs5029939	1	G			3.00E-12	European	19165918
Plasma	**TNFAIP3**	P21580	rs5029937	Lupus erythematosus systemic	**GWAS**	**T**	rs5029939	1	G			3.00E-12	European	19165918
Plasma	**TNFAIP3**	P21580	rs5029937	Selective immunoglobulin A deficiency IgAD	**GWAS**	**T**	rs2230926	1	G			1.30E-17	European	20694011

Plasma	**TNFAIP3**	P21580	rs5029937	Systemic lupus erythematosus	GWAS	**T**	rs2230926	1	G			2.00E-16	Mixed	27399966
Plasma	**TNFAIP3**	P21580	rs5029937	Rheumatoid arthritis	GWAS	**T**	rs2230926	1	G	0.3011	0.02761	5.80E-25	Mixed	24390342
Plasma	**ERBB3**	P21860	rs773116	Qualifications: college or university degree	GWAS	**A**	rs798829	1	A	0.006375	0.001142	2.40E-08	European	UKBB
Plasma	**ERBB3**	P21860	rs773116	Years of educatiol attainment	GWAS	**A**	rs798829	1	A	0.015	0.003	6.93E-09	European	27225129
Plasma	**ERBB3**	P21860	rs773116	Arm fat mass right	GWAS	**A**	rs705708	1	A	-0.01335	0.002382	2.13E-08	European	UKBB
Plasma	**ERBB3**	P21860	rs773116	Body mass index	GWAS	**A**	rs705708	1	A	-0.01555	0.002405	1.01E-10	European	UKBB
Plasma	**ERBB3**	P21860	rs773116	Impedance of arm left	GWAS	**A**	rs705708	1	A	0.0113	0.001717	4.70E-11	European	UKBB
Plasma	**ERBB3**	P21860	rs773116	Impedance of arm right	GWAS	**A**	rs705708	1	A	0.009934	0.00171	6.27E-09	European	UKBB
Plasma	**ERBB3**	P21860	rs773116	Impedance of leg left	GWAS	**A**	rs705708	1	A	0.01212	0.002217	4.63E-08	European	UKBB
Plasma	**ERBB3**	P21860	rs773116	Impedance of whole body	GWAS	**A**	rs705708	1	A	0.01197	0.001854	1.08E-10	European	UKBB
Plasma	**ERBB3**	P21860	rs773116	Self-reported hypothyroidism or myxoedema	GWAS	**A**	rs705708	1	A	0.002866	0.000519	3.40E-08	European	UKBB
Plasma	**ITPKA**	P23677	rs316617	Lymphocyte percentage of white cells	GWAS	**C**				0.02753	0.003743	1.91E-13	European	27863252
Plasma	**ITPKA**	P23677	rs316617	Neutrophil percentage of white cells	GWAS	**C**	rs1655557	1	C	-0.02631	0.003732	1.80E-12	European	27863252
Plasma	**ITPKA**	P23677	rs316617	Leg fat percentage right	GWAS	**C**	rs1655557	1	C	0.008814	0.001609	4.30E-08	European	UKBB
Plasma	**ITPKA**	P23677	rs316617	Granulocyte count	GWAS	**C**	rs1757456	1	C	-0.0219	0.003773	6.49E-09	European	27863252
Plasma	**ITPKA**	P23677	rs316617	Myeloid white cell count	GWAS	**C**	rs1757456	1	C	-0.02149	0.003783	1.34E-08	European	27863252
Plasma	**ITPKA**	P23677	rs316617	Neutrophil count	GWAS	**C**	rs1757456	1	C	-0.0229	0.003764	1.17E-09	European	27863252

Plasma	**ITPKA**	P23677	rs316617	Sum basophil neutrophil counts	GWAS	C	rs1757456	1	C	-0.02274	0.003771	1.64E-09	European	27863252
Plasma	**ITPKA**	P23677	rs316617	Sum neutrophil eosinophil counts	GWAS	C	rs1757456	1	C	-0.02212	0.003768	4.30E-09	European	27863252
Plasma	**IL1RL1**	Q01638	rs13020553	Asthma	GWAS	G	rs12470864	1	A	0.01048	0.000799	2.70E-39	European	UKBB
Plasma	**IL1RL1**	Q01638	rs13020553	Doctor diagnosed asthma	GWAS	G	rs12470864	1	A	0.01332	0.001671	1.61E-15	European	UKBB
Plasma	**IL1RL1**	Q01638	rs13020553	Self-reported asthma	GWAS	G	rs12470864	1	A	0.01072	0.000801	7.84E-41	European	UKBB
Plasma	**IL1RL1**	Q01638	rs13020553	Treatment with ventolin 100micrograms inhaler	GWAS	G	rs12470864	1	A	0.003282	0.000419	4.63E-15	European	UKBB
Plasma	**IL1RL1**	Q01638	rs13020553	Eosinophil percentage of granulocytes	GWAS	G	rs950880	1	A	0.06609	0.003663	8.60E-73	European	27863252
Plasma	**IL1RL1**	Q01638	rs13020553	Eosinophil percentage of white cells	GWAS	G	rs950880	1	A	0.06755	0.003647	1.34E-76	European	27863252
Plasma	**IL1RL1**	Q01638	rs13020553	Neutrophil percentage of granulocytes	GWAS	G	rs950880	1	A	-0.06154	0.003663	2.43E-63	European	27863252
Plasma	**IL1RL1**	Q01638	rs13020553	Crohns disease	GWAS	G	rs13001325	1	T			6.00E-11	Mixed	28067908
Plasma	**IL1RL1**	Q01638	rs13020553	Inflammatory bowel disease	GWAS	G	rs13001325	1	T			5.00E-11	Mixed	28067908
Plasma	**IL1RL1**	Q01638	rs13020553	Asthma	GWAS	G	rs13001325	1	T	0.115	0.01276	2.02E-19	European	29273806
Plasma	**IL1RL1**	Q01638	rs13020553	Eosinophil count	GWAS	G	rs1420104	1	A	0.07047	0.003649	4.31E-83	European	27863252
Plasma	**IL1RL1**	Q01638	rs13020553	Sum eosinophil basophil counts	GWAS	G	rs1420104	1	A	0.06663	0.003654	2.77E-74	European	27863252
Plasma	**IL1RL1**	Q01638	rs13020553	Wheeze or whistling in the chest in last year	GWAS	G	rs1420104	1	A	0.009566	0.001022	7.76E-21	European	UKBB

Plasma	**IL1RL1**	Q01638	rs13020553	Interleukin-1 receptor-like 1	**pQTL**	**G**				-0.5789	0.0226	6.63E-132	European	28369058
Plasma	**IL1RL1**	Q01638	rs13020553	Interleukin-1 receptor type 2	**pQTL**	**G**	rs12470864	1	A	-0.1834	0.0253	3.80E-13	European	29875488
Plasma	**IL1RL1**	Q01638	rs13020553	Interleukin-18 receptor 1	**pQTL**	**G**	rs13001325	1	T	-0.3763	0.0244	1.86E-53	European	29875488
Plasma	**TLR1**	Q15399	rs5743618	Allergic disease	**GWAS**	**A**				-0.0915	0.0067	2.73E-42	European	29083406
Plasma	**TLR1**	Q15399	rs5743618	Allergic disease asthma hay fever or eczema	**GWAS**	**A**				-0.09531	0.005924	3.00E-58	European	29083406
Plasma	**TLR1**	Q15399	rs5743618	Asthma	**GWAS**	**A**						4.00E-11	European	27182965
Plasma	**TLR1**	Q15399	rs5743618	Doctor diagnosed hayfever or allergic rhinitis	**GWAS**	**A**				-0.02414	0.002439	4.45E-23	European	UKBB
Plasma	**TLR1**	Q15399	rs5743618	Hayfever, allergic rhinitis or eczema	**GWAS**	**A**				-0.02297	0.001231	1.23E-77	European	UKBB
Plasma	**TLR1**	Q15399	rs5743618	Home area population density: Scotland large urban area	**GWAS**	**A**				-0.00415	0.000628	4.15E-11	European	UKBB
Plasma	**TLR1**	Q15399	rs5743618	No blood clot, bronchitis, emphysema, asthma, rhinitis, eczema or allergy diagnosed by doctor	**GWAS**	**A**				0.02319	0.001365	1.02E-64	European	UKBB
Plasma	**TLR1**	Q15399	rs5743618	Self-reported hayfever or allergic rhinitis	**GWAS**	**A**				-0.00576	0.00068	2.36E-17	European	UKBB
Plasma	**TLR1**	Q15399	rs5743618	Breast cancer	**GWAS**	**A**	rs6815814	1	C	-0.05827	0.008092	6.00E-13	Mixed	29059683

Plasma	**TLR1**	Q15399	rs5743618	Helicobacter pylori seroprevalence	**GWAS**	**A**	rs4833095	1	C			1.43E-18	European	23652523
Plasma	**TLR1**	Q15399	rs5743618	Pam3CSK4 induced interleukin 6 IL 6	**GWAS**	**A**	rs4833095	1	C			4.29E-26	European	23151486
Plasma	**TLR1**	Q15399	rs5743618	Asthma and hay fever	**GWAS**	**A**	rs4833095	1	C	-0.1823	0.0264	5.00E-12	European	24388013
Plasma	**KYNU**	Q16719	rs12477146	Lymphocyte count	**GWAS**	**A**	rs2008533	1	A	-0.03295	0.004686	2.04E-12	European	27863252
Plasma	**KYNU**	Q16719	rs12477146	Platelet distribution width	**GWAS**	**A**	rs2008533	1	A	0.03089	0.004678	4.02E-11	European	27863252
Plasma	**KYNU**	Q16719	rs12477146	Kynurenise	**pQTL**	**A**				-0.5017	0.0312	5.01E-58	European	29875488
Plasma	**LAYN**	Q6UX15	rs4938792	Self-reported hypertension	**GWAS**	**C**	rs542275	1	C	0.006232	0.001095	1.25E-08	European	UKBB
Plasma	**IL1RL2**	Q9HB29	rs3917265	Interleukin-1 receptor-like 2	**pQTL**	**T**				0.1909	0.0248	1.35E-14	European	29875488

Supplementary Table.2 Previously reported SNPs for eleven causal proteins are employed as genetic instruments significantly linked with the whole genome.
