# Supplementary material for: Identification of potential drug targets for allergic diseases from a genetic perspective: A mendelian randomization study
Source: Clin Transl Allergy. 2024 Apr 4;14(4):e12350. doi: 10.1002/clt2.12350 (PMC10994001; doi:10.1002/clt2.12350)
Supplement: Supplementary file 6 — Table S3 [file CLT2-14-e12350-s005.pdf]

Compound	Protein	Free energy/(kcal/mol)
Dexamethasone	LAYN	-7.89
Dronabinol	LAYN	-6.39
Ethinylestradiol	LAYN	-7.28
Calcitriol	LAYN	-6.81
Cyclosporine	TNFAIP3	-13.08
Vincristine	TNFAIP3	-9.39
Withaferin A	TNFAIP3	-6.57
Ursodeoxycholic acid	TNFAIP3	-6.44

Supplementary Table.S3 Potential drug screening.The free energy of binding is less than -5, which indicates that the small molecular compound has a stable binding with the protein.
